# Differences in the antimicrobial susceptibility profiles of *Moraxella bovis*, *M. bovoculi* and *M. ovis*

**DOI:** 10.1590/S1517-838246220140058

**Published:** 2015-06-01

**Authors:** Grazieli Maboni, Leticia T. Gressler, Julia P. Espindola, Marcelo Schwab, Caiane Tasca, Luciana Potter, Agueda Castagna de Vargas

**Affiliations:** 1 Universidade Federal de Santa Maria, Departamento de Medicina Veterinária Preventiva, Universidade Federal de Santa Maria, Santa Maria, RS, Brasil, Departamento de Medicina Veterinária Preventiva, Universidade Federal de Santa Maria, Santa Maria, RS, Brazil.; 2 Universidade Federal de Santa Maria, Departamento de Zootecnia, Universidade Federal de Santa Maria, Santa Maria, RS, Brasil, Departamento de Zootecnia, Universidade Federal de Santa Maria, Santa Maria, RS, Brazil.

**Keywords:** bacterial resistance, broth microdilution, eye disease

## Abstract

The aim of this study was to determine the differences in the antimicrobial susceptibility profiles of *Moraxella bovis*, *M. bovoculi* and *M. ovis*. Thirty-two strains of *Moraxella* spp. isolated from cattle and sheep with infectious keratoconjunctivitis were tested via broth microdilution method to determine their susceptibility to ampicillin, cefoperazone, ceftiofur, cloxacillin, enrofloxacin, florfenicol, gentamicin, neomycin, oxytetracycline and penicillin. The results demonstrated that *Moraxella* spp. strains could be considered sensitive for most of the antimicrobials tested in this study, but differences between the antimicrobial susceptibility profiles of these three *Moraxella* species were found. *M. bovis* might differ from other species due to the higher MIC and MBC values it presented.

## Introduction

Infectious keratoconjunctivitis (IK) affects cattle and sheep and is characterized by the development of conjunctivitis and corneal ulcers (Baptista, 1979). In cattle, the microorganisms involved in IK are *M. bovis* (Henson and Grumbles, 1960), *M. ovis* (Elad *et al.*, 1988) and *M. bovoculi* (Angelos *et al.*, 2007). In contrast, in sheep, the main microorganisms isolated from IK lesions are *Mycoplasma conjunctivae*, *Chlamydophila psittaci* and *M. ovis* (Elad *et al.*, 1988; Dagnall, 1994). *M. bovis* can also be isolated from sheep but it is found at a lower frequency (Libardoni *et al.*, 2012). The treatment of this disease is based on antimicrobial therapy, which should be adopted considering that it is necessary to combat two or more species of *Moraxella* present in the same lesion. However, studies addressing the antimicrobial susceptibility of *M. bovis*, *M. bovoculi* and *M. ovis* are scarce in the literature. To the best of our knowledge, this is the first study to report the antimicrobial susceptibility of *M. ovis* isolated from IK in sheep and the first study in Brazil to describe the antimicrobial susceptibility of *M. bovoculi*.

It is important to determine the antimicrobial susceptibility of *Moraxella* spp., so that if the disease occurs the best-possible treatment will be provided. In this context, the aim of this study was to determine the differences in the antimicrobial susceptibility profiles of *M. bovis*, *M. bovoculi* and *M. ovis*.

## Materials and Methods

### Samples characterization

Samples from clinical cases of infectious bovine and ovine IK occurring in southern Brazil were previously processed. These samples included different strains from the same herd, from different herds in similar locations and from different locations. A total of 32 samples were characterized as *Moraxella* spp. based on Gram staining and biochemical tests (Macfaddin, 2000). To perform molecular identification of species, DNA was extracted using the CTAB (cetyl-trimethylammonium bromide) method (Sambrook and Russel, 2001). For DNA amplification by the polymerase chain reaction (PCR), the primers ISR fo forward (5′ ACCGACGCTTATCGCAGGTCACTA-3′) and ISR reverse (5′-GTGTCGAAGCA AAATCAGGGTCGT-3′) were used. Fragments with a length of 650 bp corresponding to *M. bovis* and those with a length of 600 bp corresponding to *M. bovoculi* and *M. ovis* were observed (Angelos and Ball, 2007). For the differentiation of *Moraxella* species, the enzyme *Rsa*I was used, which only cleaves the amplified DNA sequence from *M. bovoculi* (600 bp) at a single restriction site, resulting in two fragments of approximately 150 and 450 bp. This enzyme does not cleave the DNA fragment of *M. bovis* (650 bp) or *M. ovis* (600 bp) (Angelos and Ball, 2007). Ten strains of *Moraxella* spp. were identified as *M. bovis*, 11 as *M. bovoculi* derived from cattle, and 11 as *M. ovis* strains isolated from sheep (Table 1).

**Table 1:** Identification, origin and year of isolation of *Moraxella bovis*, *M. bovoculi* and *M. ovis* strains.

*Moraxella bovis*		*Moraxella bovoculi*		*Moraxella ovis*	
		
Identification	Origin (year of isolation)	Identification	Origin (year of isolation)	Identification	Origin (year of isolation)
SB 24/90	São Martinho da Serra, RS (1990)	SB156/96	Lavras do Sul, RS (1996)	SB567/05	Santa Maria, RS (2005)
TORRES	Pelotas, RS (−)	SB163/03	Formigueiro, RS (1993)	SB06/08	Caçapava do Sul, RS 2008)
SBP111/12 T939	Dom Pedrito, RS (2012)	SB15/13	Caçapava do Sul, RS (2013)	SB326/07	Santa Maria, RS (2007)
SBP 111/12 889T	Dom Pedrito, RS (2012)	Alegrete	Alegrete, RS (−)	SB07/08	São Sepé, RS (2008)
SBP111/12 (3)	Dom Pedrito, RS (2012)	SB150/02 n°5	Tupanciretã, RS (2002)	SB07/13 n°3	São Sepé, RS (2013)
SBP 111/12 (2)	Dom Pedrito, RS (2012)	Itapuã	Itapuã, RS (−)	SB07/13 n°2	São Sepé, RS (2013)
SB 82/92	Dilermando de Aguiar, RS (1992)	SB296/07	Santa Maria, RS (2007)	SB07/13 n° 1	São Sepé, RS (2013)
2439	Pelotas, RS (−)	Viviane	Pelotas, RS (−)	Água doce	(−)
147	Pelotas, RS (−)	Jackson	(−)	Nunes	(−)
05/329	Pelotas, RS (−)	SB57/12 (8127)	Minas Gerais (2012)	SB247/92	Santa Maria, RS (1992)
ATCC *M. bovis*	Number: 10900	SB15/10	Pelotas, RS (2010)	SB249/92 n°6	Cruz Alta, RS (1992)
		ATCC *M. bovoculi*	Number: BAA1259	ATCC *M. ovis*	Number: 19575

(−) Information not available.

RS: Rio Grande do Sul, Brazil.

### Broth microdilution method

The MIC (minimal inhibitory concentration) and MBC (minimal bactericidal concentration) were investigated for ampicillin, gentamicin, neomicin, penicillin, cefoperazone, ceftiofur, cloxacillin, enrofloxacin, florfenicol and oxytetracycline. The MIC was determined in 96-well microplates, in which 100 μL of the bacterial inoculum (10^5^ cfu/mL) was added to 100 μL of Muller Hinton broth (cation adjusted with 20 g/L of calcium and 10 g/L of magnesium), in this volume, 12 different concentrations of each antimicrobial agent were diluted for 32 mg/mL to 0.015 mg/mL. All strains were analyzed in triplicate along with reference strains (*M. bovis* ATCC 10900, *M. bovoculi* ATCC BAA1259 and *M. ovis* ATCC 19575). *Staphylococcus aureus* ATCC 25923 was used as the standard quality control strain. Finally, the microplates were incubated under aerobic conditions at 35 °C for 24 h (CLSI, 2013).

To determine the MBC, 10 μL of each antimicrobial dilution corresponding to each strain, equal or higher than the MIC value, was transferred to Muller-Hinton agar plates and followed by incubation at 35 °C for 24 h.

As no standardized criteria for the interpretation of sensitivity exist for *Moraxella* spp., breakpoints established for Gram-negative pathogens related with bovine respiratory disease were used. No established breakpoints were available for cloxacillin, cefoperazone and neomycin (CLSI, 2013). The MIC was defined as the lowest concentration of antibiotics that completely inhibited growth, and the MIC_50_ and MIC_90_ were defined as the lowest concentrations of antibiotics capable of inhibiting the growth of 50% and 90% of the *Moraxella* spp. isolates, respectively. The MBC_50_ and MBC_90_ have been defined as the lowest concentrations of antimicrobial agents at which no bacterial growth is evident in 50% and 90% of isolates, respectively (CLSI, 2013).

### Statistical analysis

The MIC and MBC values were evaluated via the Kruskal-Wallis test and subjected to the calculation of position measurements. When differences between species of *Moraxella* were identified, the Bonferroni test was applied to compare the modal MIC values.

## Results and Discussion

The results of the broth microdilution method for the 32 strains of *Moraxella* spp. are shown in Table 2. Generally, the MIC and MBC values were similar, indicating that the same concentrations of the antimicrobial agents were able to both inhibit bacterial growth and kill the microorganism (Table 2). Although there are no guidelines to define MICs for *Moraxella* spp, interpretative criteria derived from other pathogens have been proposed (Angelos *et al.*, 2011). For instance, the critical breakpoints for determining ampicillin, ceftiofur, enrofloxacin, florfenicol, gentamicin, penicillin and oxytetracycline efficacy against respiratory pathogens of cattle (*Pasteurella multocida*, *Mannheimia haemolytica*, and *Haemophilus somnus*) could also represent interpretative data of *M. bovis*, *M. bovoculi* and *M. ovis* susceptibility to these antimicrobials. According to these interpretative criteria, most *Moraxella* spp. strains were considered susceptible to most antimicrobials, however some strains were considered resistant to penicillin and oxytetraciline, such as *M. bovis* strains that showed 40% (4/10) of resistance to penicillin and 20% (2/10) of resistance to oxytetracycline (> 2). Other studies found *M. bovis* strains, isolated in the United States, resistant to gentamicin and oxytetracicline (Shryock *et al.*, 1998). Moreover, *M. bovis* strains resistant to erythromycin were also reported in South America (Conceição *et al.*, 2004).

**Table 2:** Minimum inhibitory and bactericidal concentrations (MIC_50_, MIC_90_, MBC_50_ and MBC_90_) modal/MIC and resistance of *Moraxella bovis*, *M. bovoculi* and *M. ovis.*

	*Moraxella bovis*					

Antimicrobial agents	MIC_50_	MIC_90_	MBC_50_	MBC_90_	Modal-MIC (μg/mL)	% resistence
Ampicillin	0.125	2	0.125	2	0.015	0.0
Cefoperazone	4	32	4	32	32	†
Ceftiofur	0.125	1	0.125	2	0.125	0.0
Cloxacillin	2	> 32	2	> 32	32	†
Enrofloxacin	0.03	0.06	0.06	0.06	0.0156	0.0
Florfenicol	1	1	1	1	1	0.0
Gentamicin	0.125	1	0.125	1	0.0156	0.0
Neomycin	0.5	1	1	2	1	†
Oxitetracycline	0.5	8	0.25	2	0.25	20.0
Penicillin	0.25	2	0.25	2	0.125	40.0

	*Moraxella bovoculi*					
	
Antimicrobial agents	MIC_50_	MIC_90_	MBC_50_	MBC_90_	Modal-MIC (μg/mL)	% resistence

Ampicillin	< 0.015	< 0.015	< 0.015	< 0.015	0.0156	0.0
Cefoperazone	0.25	4	1	4	0.25	†
Ceftiofur	< 0.015	0.03	< 0.015	0.03	0.0156	0.0
Cloxacillin	0.06	> 32	0.125	> 32	0.0625	†
Enrofloxacin	0.03	1	0.06	0.125	0.0312	0.0
Florfenicol	0.5	1	1	1	0.25	0.0
Gentamicin	0.5	1	0.5	2	0.5	0.0
Neomycin	1	2	2	2	1	†
Oxitetracycline	0.5	12	2	2	0.25	0.0
Penicillin	< 0.015	< 0.015	< 0.015	< 0.015	0.0156	9.0

	*Moraxella ovis*					
	
Antimicrobial agents	MIC_50_	MIC_90_	MBC_50_	MBC_90_	Modal-MIC (μg/mL)	% Resistence

Ampicillin	< 0.015	< 0.03	< 0.015	< 0.015	0.015	0.0
Cefoperazone	4	16	8	16	2	†
Ceftiofur	0.06	0.25	0.06	0.25	0.015	0.0
Cloxacillin	0.5	1	2	4	1	†
Enrofloxacin	0.06	0.125	0.25	0.25	0.125	0.0
Florfenicol	1	2	1	2	0.5	0.0
Gentamicin	0.5	1	1	1	0.5	0.0
Neomycin	1	2	1	2	1	†
Oxitetracycline	0.5	4	2	8	0.5	9.0
Penicillin	< 0.015	0.5	< 0.015	0.5	0.0156	18.0

† Cannot be calculated; no defined breakpoints.

According to the interpretative criteria used in this study, 9% (1/11) of *M. bovoculi* strains could be considered resistant to penicillin. A previous study reported similar results for *M. bovoculi*, indicating higher resistance to penicillin (12.3%) in comparison with other tested antimicrobials (Angelos *et al.*, 2011). The data reported in the veterinary literature regarding the susceptibility of *Moraxella* spp. predate the description of *M. bovoculi*, consequently, it is mainly available from studies involving *M. bovis*. (Webber *et al.*, 1982; Shryock *et al.*, 1998; Zielinski *et al.*, 2002; Conceição *et al.*, 2004). In this way, the antimicrobial susceptibility of *M. bovoculi* was only evaluated after the recent description of this species (Angelos *et al.*, 2007), in a study in the United States (Angelos *et al.*, 2011). Thus, this is the second study evaluating the susceptibility profile of *M. bovoculi*.

Some *M. ovis* strains examined in this study could be considered resistant to oxytetracycline (9% – 1/11) and penicillin (18% – 2/11). The susceptibility data for *M. ovis* available in the literature are scarce; the only study addressing the antimicrobial susceptibility of *M. ovis* examined isolates from cattle and reported resistance only for erythromycin (Catry *et al.*, 2007). One reason for this lack of information may be that this pathogen is not the primary agent involved in the etiology of the disease in sheep (Dagnall, 1994). It is important to note that the present report is the first to describe the susceptibility of *M. ovis* derived from sheep, and this etiological agent should also be controlled by antibiotics, especially to avoid exacerbation of lesions primarily caused by *M. conjunctivae* and *C. pscittaci* (Dagnall, 1994).

Oxytetracycline is usually the first choice for antimicrobial treatment of IK (Alexander, 2010). The MIC_50_ and MIC_90_ values obtained for oxytetracycline are presented in Table 2. The previously reported MIC values for oxytetracycline for *M. bovis* (Shryock*et al*., 1998), *M. bovoculi* (Angelos *et al.*, 2011), and *M. ovis* (Catry *et al.*, 2007) are lower than the values reported in this study. Although oxytetracycline is widely used in the treatment of this disease, there are only two reports of resistance of *M. bovis* to this drug in the literature (Shryock *et al.*, 1998; Senturk *et al.*, 2007). In the present study, 20% of the *M. bovis* strains and 9% of the *M. ovis* strains could be considered resistant to oxytetracycline. These results may suggest that the indiscriminate use of oxytetracycline over the years can be related with the selection of *Moraxella* spp. strains resistant to this drug.

We observed significantly high MIC values (16 μL/mL to > 32 μL/mL) for cloxacillin. Susceptibility of *M. bovis* for cloxacillin was reported (Webber *et al.*, 1982), and strains displaying high MIC values, similar to those found in the present study, were considered resistant. The MIC_90_ values for florfenicol obtained for *M. bovis*, *M. bovoculi* and *M. ovis* were 1 μL/mL, 1 μL/mL and 2 μL/mL, respectively. A previous study found 3.5% of resistance for florfenicol among *M. bovoculi* strains (Angelos *et al.*, 2011). MIC_90_ values for florfenicol, similar to those found in this study, were reported for *M. bovis* strains isolated in Argentina (Zielinski *et al.*, 2002) and *M. ovis* strains isolated in Belgium (Catry *et al.*, 2007). Similar to oxytetracycline, florfenicol has been reported to be an effective treatment option for combating bovine IK (Gocke *et al.*, 2002; Angelos *et al.*, 2011), especially in cases where *M. bovis* is resistant to tetracycline antibiotic class (Angelos *et al.*, 2000).

The statistical analysis showed that the highest modal values of MIC occurred among the *M. bovis* strains. In contrast, *M. bovoculi* displayed the lowest modal values of MIC (Table 2). Based on the results obtained using the broth microdilution it can be suggested that there is difference (p < 0.05) between the antimicrobial profile of *M. bovis* and those of *M. bovoculi* and *M. ovis*. Further studies are necessary to determine the reason that higher concentrations of antimicrobials are required to achieve inhibition of *M. bovis*. According to the interpretative criteria used, the in vitro results demonstrate that the three *Moraxella* species showed the best susceptibility profile for ampicillin, ceftiofur, enrofloxacin, florfenicol and gentamicin.
